# MD-Transformer: Multimodal Integration of ProtBERT Embeddings and Physicochemical Descriptors for Protein–Protein Interface Residue Prediction

**DOI:** 10.3390/ijms27135848

**Published:** 2026-06-29

**Authors:** Jiahui Yang, Jihua Feng, Yuting Zhang, Zhongxing Chen

**Affiliations:** 1 School of Electrical and information Technology, Yunnan Minzu University, Kunming 650500, China; 24214038160030@ymu.edu.cn (J.Y.); 24214038160029@ymu.edu.cn (Y.Z.); 23214038160007@ymu.edu.cn (Z.C.); 2 Yunnan Key Laboratory of Unmanned Autonomous Systems, Kunming 650500, China

**Keywords:** protein–protein interaction, interface residue prediction, multimodal learning, ProtBERT, physicochemical descriptors, transformer, solvent-accessible surface area, cross-modal attention

## Abstract

Accurate prediction of *protein–protein interaction* (PPI) interface residues is essential for understanding molecular recognition and supporting structure-guided design. To integrate contextual sequence representations with structure-related physicochemical information, we propose a multimodal framework termed MD-Transformer. The model combines residue-level ProtBERT embeddings with physicochemical descriptors, including B-factor, *solvent-accessible surface area* (SASA), and hydrophobicity. A hybrid fusion module first aligns heterogeneous features, followed by Transformer encoding and cross-modal attention for multimodal integration. Using the DB5.5 benchmark, physicochemical descriptors were Z-score normalized exclusively with training-set statistics. Under the complex-level split protocol (Official A), MD-Transformer achieved an AUPRC of 0.564, outperforming the ablation model without physicochemical descriptors by 0.159 and reducing false-positive predictions on exposed non-interface residues. Under the homology-aware split protocol (Official B v1), the model maintained an AUPRC of 0.480 and an MCC of 0.242, indicating retained predictive capability under reduced sequence similarity constraints. Under the same aligned evaluation workflow, PeSTo achieved an AUPRC of 0.264. Further SASA-stratified analyses identified SASA as a major contributor to suppressing false-positive predictions across residue exposure environments, while also revealing a precision-recall trade-off in highly exposed residues. These results suggest that contextual sequence representations and residue-level physicochemical descriptors provide complementary predictive signals.

## 1. Introduction

*Protein–protein interactions* (PPIs) underlie essential biological processes, including signal transduction and immune recognition, and play important roles in disease mechanisms at the molecular level [1,2,3]. PPI interface residue prediction aims to identify binding-relevant residues on protein surfaces. Compared with binary PPI prediction, residue-level prediction provides finer spatial information. However, accurate prediction remains difficult because of severe class imbalance, complex physicochemical interactions, and long-range residue dependencies [4]. Accurate identification of interface residues is critical for antibody engineering, peptide inhibitor design, and the interpretation of disease-associated mutations affecting protein recognition. Recent deep learning approaches have improved residue-level PPI prediction by integrating sequence-derived, structural, and geometric representations [5].

Traditional approaches largely relied on hand-crafted features such as sequence conservation, solvent accessibility, and electrostatic properties [4,6]. Although these descriptors are biologically interpretable, they have limited capacity to capture deep evolutionary semantics and complex interactions among heterogeneous biological signals. Recent advances in protein language models, including ESM [7,8] and ProtBERT [9], have demonstrated strong performance by learning contextual sequence representations from large-scale protein corpora. More broadly, foundation-model-based approaches in bioinformatics have further highlighted the potential of large-scale representation learning for downstream structural and functional prediction tasks [10]. However, residue-level interface prediction is strongly influenced by structural context and surface geometry. Sequence-based language models may assign high scores to exposed non-interface residues, thereby increasing false-positive predictions. Structure-based methods such as ScanNet [11] and DeepRank-GNN [12] incorporate local geometric information and improve interface characterization, although their performance may depend on structural quality and data availability. In addition, structural descriptors and language-model embeddings typically reside in different feature spaces, and the lack of effective alignment can limit the complementary benefit of multimodal integration [13,14,15].

Interface formation is shaped by conformational flexibility, solvent exposure, and physicochemical compatibility [11,13]. At the residue level, the B-factor can provide an indirect measure of local flexibility in experimentally determined structures [16,17]. However, its interpretation depends on the source and refinement procedure of the input structure. SASA reflects residue exposure [18]. Hydrophobicity describes the tendency of residues to be buried at interfaces and contributes to hydrophobic-driven interactions [19]. These descriptors are readily obtainable from structural data and may improve specificity by suppressing false-positive predictions on surface residues. On this basis, we propose a multimodal deep learning framework, the Multi-Descriptor Transformer (MD-Transformer). The model takes residue-level ProtBERT embeddings [9] as the sequence modality and combines them with residue-level physicochemical descriptors. A hybrid fusion layer first aligns heterogeneous features, followed by a Transformer encoder that captures long-range contextual dependencies. Cross-modal attention is then applied to integrate sequence semantics with physicochemical information [20,21]. This design aims to improve discrimination between interface and non-interface residues while preserving long-range contextual dependencies.

To reduce performance inflation caused by overlap at the complex level or among homologous sequences [22], we constructed a layered evaluation framework based on the Protein–Protein Docking Benchmark 5.5 (DB5.5) [23]. In addition to the complex-level split (Official A), we introduced a homology-aware split (Official B v1) to evaluate model behavior under stronger homology constraints. We further characterized strict split behavior using CD-HIT [24] and MMseqs2 [25] clustering, and performed supplementary evaluation on PDB structures released after DB5.5. Because temporal separation alone does not guarantee sequence independence, the external set was further filtered by removing complexes with chain-level homology to the Official A training set, resulting in a homology-filtered external evaluation.

In this study, the main contribution is not the introduction of a generic Transformer backbone, but the task-specific integration and evaluation of contextual PLM embeddings with interpretable residue-level physicochemical descriptors for interface-residue prediction. We specifically examine whether a small set of structure-derived descriptors can reduce exposed-surface false positives when combined with frozen sequence representations under leakage-aware evaluation protocols.

The main contributions of this work are summarized as follows:

(1) We propose MD-Transformer, a physicochemical-guided multimodal framework for residue-level PPI interface prediction. The model integrates ProtBERT residue embeddings with B-factor, SASA, and hydrophobicity through a hybrid fusion layer and a physicochemical cross-modal attention module, rather than relying on simple feature concatenation.
(2) We establish a leakage-aware and scenario-aware evaluation workflow, including training-set-only normalization, complex-level and homology-aware splitting, validation-selected thresholds fixed for test evaluation, strict-split stress tests, homology-filtered external evaluation, and unbound-structure evaluation.
(3) Through ablation studies, error analysis, and SASA-stratified evaluation, we show that physicochemical descriptors, particularly SASA, reduce false-positive predictions on exposed non-interface residues, while also revealing a precision-recall trade-off across residue exposure environments.

The intended use case of MD-Transformer is to prioritize likely interface residues when only individual protein structures, unbound protein structures, or predicted subunit structures are available, rather than when the bound complex structure has already been experimentally determined.

## 2. Results

Because the evaluation workflow contains multiple protocols with different splitting assumptions and intended interpretations, we summarize the full evaluation design in Table S10 before reporting model performance.

### 2.1. Performance Under the Official A Setting

MD-Transformer was evaluated under the Official A protocol, which uses the standard complex-level split of DB5.5. In this setting, all chains from a given protein complex are assigned to a single subset, namely training, validation, or test, thereby preventing the same complex from contributing to more than one subset. This split evaluates performance on held-out complexes but does not explicitly control sequence similarity between chains from different complexes. Official B v1 uses the same DB5.5 benchmark and preserves this complex-level separation, but additionally groups sequence-similar chains before splitting, providing a more stringent homology-aware evaluation.

Under Official A, the model achieved an AUROC of 0.783 and an AUPRC of 0.564. For threshold-dependent metrics, decision thresholds were scanned on the validation set to maximize the F1-score. The selected threshold of 0.56 was then fixed and applied to the test set. At this threshold, the model achieved an MCC of 0.366, a precision of 0.467, a recall of 0.596, and an F1-score of 0.524. Table 1 summarizes the results under the two DB5.5 evaluation protocols.

### 2.2. Ablation and Contribution of Physicochemical Features

To quantify the contribution of physicochemical descriptors, we performed an ablation by setting these features to zero. On the Official A test set, AUPRC decreased from 0.564 to 0.405, corresponding to a reduction of 0.159, while MCC decreased from 0.366 to 0.158. Using the fixed threshold of 0.56, the ablation model retained high recall at 0.976 but showed low precision at 0.251. Removing physicochemical descriptors led to systematic overprediction of interface residues, particularly on exposed protein surfaces.

**Figure 1:**
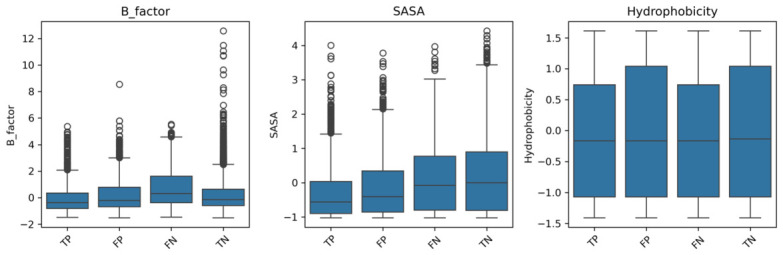
Physicochemical Feature Distributions across Prediction Outcome Types under Official A at a Fixed Threshold. Physicochemical descriptors were normalized using training-set-only Z-score statistics. The decision threshold was selected by maximizing validation F1-score (t = 0.56) and fixed for the test set. Test residues are grouped into TP, FP, FN, and TN, and the distributions of B-factor, SASA, and hydrophobicity are compared.

At the same threshold, predictions on the Official A test set comprised 1632 true positives, 1860 false positives, 1104 false negatives, and 7442 true negatives among 12,038 residues in total. We then compared the distributions of SASA, B-factor, and hydrophobicity across TP, FP, FN, and TN groups. These descriptors exhibited distinct distributions across prediction outcome categories, as shown in Figure 1. Clear distributional separation was evident, most notably for SASA, suggesting that solvent exposure contributes to suppressing false-positive predictions on non-interface surface residues.

To evaluate the apparent overprediction observed in structural visualization, we compared the predicted number of interface residues with the ground-truth interface count at both complex and chain levels under the Official A protocol, the model predicted 3492 interface residues compared with 2736 ground-truth interface residues, corresponding to a predicted-to-true ratio of 1.276. At the complex level, 23 of 26 complexes showed overprediction, and a one-sided exact sign test indicated that overprediction occurred more frequently than underprediction (*p* = $4.4\times 10^{-5}$; Table S12). Under Official B v1, overprediction was more pronounced, with a predicted-to-true ratio of 1.942. These results indicate that the model tends to overpredict interface residues at the validation-selected threshold, particularly under stronger homology constraints. This behavior is consistent with threshold selection based on validation F1-score, which balances precision and recall but does not explicitly calibrate the total number of predicted interface residues.

To further examine whether the contribution of SASA reflected improved discrimination or a bias toward less exposed residues, we stratified Official A test residues into four quartiles according to SASA and compared precision-recall behavior across exposure environments. As shown in Table S8 and Figure S7, the md = 0 ablation produced consistently high false-positive rates across all SASA quartiles, ranging from 0.815 to 0.901. In contrast, the full model reduced the false-positive rates to 0.216, 0.281, 0.199, and 0.120 from the lowest to highest SASA quartiles, respectively. Precision also improved across all quartiles. However, recall decreased compared with the md = 0 ablation, particularly in the highest-SASA quartile. Thus, SASA mainly constrained overprediction on exposed non-interface residues and improved specificity, rather than uniformly increasing sensitivity to all interface residues.

**Figure 2:**
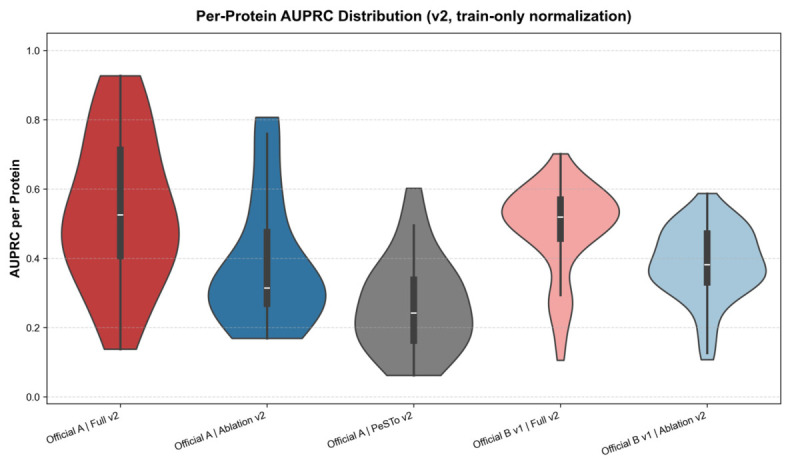
Per-Complex AUPRC Distributions under Official A and Official B v1. Per-complex AUPRC is computed for each complex and compared across the MD-Transformer full model, the ablation model, and realigned PeSTo under different protocols. For the ablation model, labels are aligned to the full model using within-chain residue order. This figure assesses whether the performance gain is consistent across complexes rather than driven by a few samples.

At the per-complex level, paired Wilcoxon tests based on AUPRC demonstrated that the full model significantly outperformed both the ablation model and PeSTo. Compared with the ablation model, the full model achieved a median AUPRC improvement of 0.116 with a *p*-value of $4.57\times 10^{-6}$. Relative to PeSTo, the median improvement reached 0.205 with a *p*-value of $7.45\times 10^{-8}$. Detailed statistical results are provided in Table S1. As shown in Figure 2, the full model achieved higher per-complex AUPRC values. Performance gains were observed across most complexes rather than being driven by a few samples. When the interface distance cutoff was changed from 6 Å to 5 Å, the full model remained superior to the ablation model, as shown in Table S2, which demonstrated that the main conclusions were robust to the choice of interface definition.

### 2.3. Generalization Under the Official B v1 Setting

Under the more stringent Official B v1 protocol, both complex-level separation and chain-level similarity constraints were imposed. MD-Transformer achieved an AUROC of 0.700 and an AUPRC of 0.480. Using the same validation-based threshold selection procedure, the selected threshold was 0.48, yielding an MCC of 0.242 and an F1-score of 0.489 on the test set, as summarized in Table 1.

Performance decreased compared with Official A, as expected, because the homology-aware split reduces the chance that test chains share close sequence patterns with training chains. Despite the stricter homology constraints, MD-Transformer remained predictive and outperformed the ablation model. The ablation model achieved an AUPRC of 0.322 and an MCC of 0.079, indicating that the contribution of physicochemical descriptors was still observed under stronger homology constraints.

Multi-seed experiments showed only minor variation across repeated runs with fixed data splits, as reported in Table S3. This result suggests that the Official B v1 performance was not driven by random initialization and supports the stability of the main findings.

### 2.4. Benchmarking Context and Comparison with Baselines

On the Official A test set, we aligned PeSTo outputs to the residue order using within-chain position indices and computed threshold-independent metrics on the commonly covered residues. All 12,038 residues across 26 test complexes were successfully matched, as summarized in Table S4, so AUROC and AUPRC were computed on the same residue set without bias from alignment loss.

**Figure 3:**
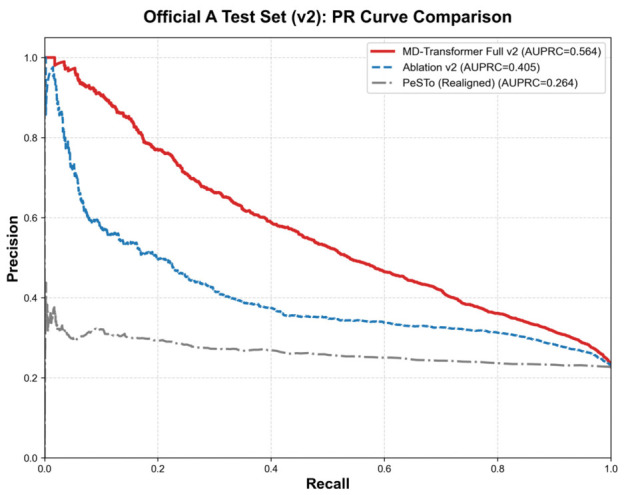
Precision-Recall Curves on the Official A Test Set. Precision-Recall curves were evaluated on the Official A test set using train-only normalization. MD-Transformer achieved an AUPRC of 0.564, compared with 0.405 for the ablation model and 0.264 for PeSTo. All reported values correspond to threshold-independent evaluation metrics.

Under this setting, PeSTo achieved AUPRC of 0.264, which was lower than the value of 0.564 obtained by the MD-Transformer full model. The precision-recall curves are shown in Figure 3. PeSTo produces uncalibrated scores on a numeric scale different from the probability outputs of our model. Therefore, only the threshold-independent metrics AUROC and AUPRC were used for external comparison, whereas threshold-dependent metrics such as MCC, precision, recall, and F1-score were not directly compared. The comparison was intended only to provide a reference under the same evaluation workflow rather than to reproduce the original PeSTo training setting.

For comparison with protein language model-based predictors, we added an ESM2-t12-only baseline using residue-level embeddings from facebook/esm2_t12_35M_UR50D. As shown in Table S11, ESM2-t12-only improved over the ProtBERT-only baseline on Official A, achieving an AUPRC of 0.543 compared with 0.503. Nevertheless, the full MD-Transformer achieved higher AUPRC and MCC than both PLM-only baselines. Under Official B v1, the full model also outperformed ESM2-t12-only, suggesting that physicochemical descriptors provide complementary information beyond sequence representations under homology-aware evaluation.

In addition to direct numerical baselines, we expanded the methodological comparison in Table S9 to include representative recent structure-aware and protein language model-based approaches, including ScanNet, DeepRank-GNN, PeSTo, ProtBERT-only, and ESM2-t12-only baselines. Because these methods differ in task formulation, input requirements, and output granularity, direct numerical comparison was restricted to baselines that could be evaluated under the same DB5.5 residue-level label definition.

### 2.5. Structural Analysis of Strict Splits

To examine how strict homology clustering affects splitting on DB5.5, we constructed strict split schemes as stress tests using CD-HIT and MMseqs2 at a 40% similarity threshold. Combining complex-level and strict homology constraints produced large connected components in the graph structure. Under CD-HIT 40 and MMseqs2 40, the largest clusters contained 262 and 246 chains, respectively, causing the test-set proportion to deviate from the target 10% and reach 40.4% and 37.8%. Summary statistics are presented in Table 2. Additional structural statistics, including the number of covered chains, homology clusters, and largest cluster sizes, are provided in Table S5.

In DB5.5, combining complex-level and strict homology constraints caused dataset connectivity to dominate the split, leading to imbalanced train/test distributions. We used strict splits mainly for structural stress testing and dataset characterization rather than as the primary performance reporting protocol. Cluster size distributions are shown in Figures S1–S4.

### 2.6. Structural Visualization

**Figure 4:**
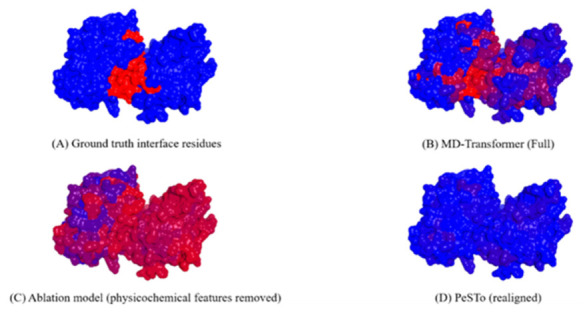
Structural Visualization of Interface Residue Prediction for Complex 1AVX under Official A. (**A**) Ground-truth interface residues; (**B**) MD-Transformer full model; (**C**) ablation model (physicochemical features set to zero); (**D**) PeSTo (realigned). Predicted probabilities were written into the B-factor field and visualized in PyMOL, with color indicating increasing interface probability.

To inspect predictions in 3D, we visualized selected Official A test complexes using PyMOL (Version 3.1.7.2) [26]. Figure 4 illustrates complex 1AVX, in which MD-Transformer yields probability patterns that are more localized to the annotated interface, whereas the ablation model shows a more diffuse distribution across the surface. To avoid reliance on a single example, two additional complexes (1D6R and 1OPH) are shown in Figures S5 and S6 and exhibit similar trends. Overall, these structural visualizations are consistent with the quantitative results and support improved interface localization by incorporating physicochemical descriptors. Because visual inspection can suggest apparent overprediction, we further quantified predicted interface-residue counts at complex and chain levels; the statistical analysis is reported in Table S12.

### 2.7. Homology-Filtered External Evaluation

We next evaluated the trained models on external-filtered, the homology-filtered external dataset described in Section 4.1.4. This dataset was derived from external-raw by removing complexes with detectable chain-level homology to the Official A training set under the ≥40% sequence identity and ≥80% query coverage criterion.

As shown in Table 3, the full MD-Transformer achieved an AUROC of 0.711, an AUPRC of 0.505, and an MCC of 0.271 on external-filtered using the validation-selected threshold of 0.56. The full model outperformed both the ablation model and the ProtBERT-only baseline in AUPRC and F1-score. Compared with the ablation model, the full model markedly reduced the false-positive rate from 0.716 to 0.365, indicating that physicochemical descriptors continued to improve specificity after homology filtering. The ProtBERT-only baseline showed lower performance on external-filtered than on external-raw, supporting the concern that the original external-raw evaluation was influenced by homology overlap. After homology filtering, MD-Transformer retained higher AUPRC and F1-score than the ablation and ProtBERT-only baselines.

### 2.8. Evaluation on DB5.5 Unbound Test Structures

To further evaluate a practical prediction scenario in which the bound complex structure is unavailable, we constructed an additional unbound test set from the DB5.5 Official A test complexes. In this experiment, the unbound receptor and ligand structures were used as model input, while the ground-truth interface labels were retained from the corresponding bound complexes.

As shown in Table 4, the full MD-Transformer achieved an AUROC of 0.721 and an AUPRC of 0.479 on the unbound test set. Compared with the md = 0 ablation, the full model substantially reduced the false-positive rate from 0.859 to 0.200 and reduced the predicted positive ratio from 0.885 to 0.264. This result indicates that physicochemical descriptors still helped constrain overprediction when unbound structures were used as input.

The ProtBERT-only baseline achieved the same reported AUROC and showed slightly higher AUPRC, MCC, and F1-score, together with a lower false-positive rate, than the full model. This suggests that sequence representations may transfer more stably under bound-to-unbound structural changes, whereas physicochemical descriptors extracted from unbound conformations may introduce distributional shifts relative to bound-state training data.

## 3. Discussion

### 3.1. Complementarity and Roles of Multimodal Features

Ablation analysis showed that B-factor, SASA, and hydrophobicity consistently improved performance under both Official A and Official B v1, suggesting that predictive performance was not driven solely by sequence semantics. At a fixed threshold, the model without physicochemical inputs displays a “high-recall, low-precision” pattern, consistent with an increased tendency to label non-interface surface residues as interface residues. In contrast, the full model improves precision while maintaining recall, leading to higher MCC and F1-score. This observation agrees with the physical interpretation of these descriptors in describing residue exposure and local microenvironments.

Baseline and architectural variant comparisons showed that models using only sequence embeddings or only physicochemical descriptors consistently underperformed relative to the full model, as summarized in Table S6. Variants based on simple concatenation or without cross-modal attention also remained less effective, suggesting that interactions between contextual sequence representations and physicochemical descriptors were important for accurate interface recognition.

Single-feature ablation analysis further indicated that SASA had the strongest influence on suppressing surface-related false positives, whereas removal of B-factor or hydrophobicity resulted in comparatively smaller performance changes, as shown in Table S7. Previous structural studies have shown that exposed surface regions may share physicochemical characteristics with interaction interfaces, which complicates residue-level discrimination [27]. The SASA-stratified analysis in Table S8 and Figure S7 further showed that the full model reduced false-positive rates across all exposure quartiles, with particularly strong reductions in highly exposed residues. However, this improvement in specificity was accompanied by reduced recall, especially in the highest-SASA quartile. Therefore, the effect of SASA should not be interpreted as simply making the model more sensitive to interface residues or as a simple bias toward buried regions. Rather, SASA appears to function primarily as a specificity-enhancing feature that calibrates predictions across residue exposure environments and suppresses overprediction on broadly exposed non-interface surfaces.

Analysis of predicted interface counts further revealed that the full model still predicted more interface residues than the ground truth at the validation-selected threshold, although this effect was less severe than the diffuse surface overprediction observed after removing physicochemical descriptors. This indicates that physicochemical descriptors improve specificity but do not fully calibrate the predicted interface size.

### 3.2. Generalization Under Homology Constraints

Official B v1 introduces chain-level similarity constraints in addition to the complex-level split, providing a more conservative setting for evaluating cross-cluster generalization. Hidden homology overlap and data leakage have been shown to inflate performance estimates in biological machine learning, particularly in sequence-related prediction tasks [28]. In this context, Official B v1 provides an important assessment of model behavior under reduced sequence similarity.

As summarized in Table 1, performance under Official B v1 decreased compared with Official A, which was expected given the stronger homology constraints. Nevertheless, MD-Transformer retained predictive capability, achieving an AUPRC of 0.480 and an MCC of 0.242. The ablation model also showed reduced performance under this setting, indicating that the contribution of physicochemical descriptors was not limited to the less restrictive Official A split. Multi-seed experiments showed only minor variation across repeated runs under fixed data splits, as reported in Table S3, further supporting the stability of the main conclusions.

The original external-raw evaluation was designed as a supplementary temporal test on structures released after DB5.5. However, MMseqs2 auditing indicated homology overlap with the Official A training set, and the strong performance of the ProtBERT-only baseline on external-raw suggested that part of the apparent external performance may have been influenced by this overlap. To address this issue, we constructed external-filtered by removing any external complex containing a chain with ≥40% sequence identity and ≥80% query coverage to the training chains. After filtering, 10 complexes with 46 chains and 9000 residues were retained for evaluation.

On external-filtered, the full model achieved higher AUPRC, MCC, and F1-score than both the ablation model and the ProtBERT-only baseline, as shown in Table 3. In contrast to external-raw, where the ProtBERT-only baseline performed strongly, its performance decreased after homology filtering. This finding supports the concern that external-raw performance was partly affected by homology overlap. Importantly, the full multimodal model retained an advantage under reduced homology overlap, suggesting that physicochemical descriptors provide complementary information beyond sequence similarity alone. The reduced false-positive rate of the full model compared with the ablation model further indicates that these descriptors continued to improve specificity in the filtered external setting.

Because external-filtered contains only 10 complexes, we interpret this experiment as supplementary homology-filtered external validation rather than as a large-scale independent benchmark. Therefore, conclusions regarding cross-homology generalization rely primarily on Official B v1, while external-filtered provides additional evidence that the model advantage is retained after removing direct sequence-level overlap with the training set.

### 3.3. Impact of Dataset Connectivity on Strict Splits

Strict split analyses based on CD-HIT and MMseqs2 show that, in DB5.5, combining strict homology constraints with complex-level constraints can yield very large connected components, which in turn shifts the train/validation/test proportions as summarized in Table 2. This structural property suggests that strict generalization assessment should consider not only similarity thresholds but also dataset connectivity, which can constrain feasible splits and affect data balance. Reproducibility and evaluation reliability are critical in biological machine learning, especially when benchmark datasets contain hidden structural or evolutionary dependencies [29]. Accordingly, we used Official A as the primary protocol for comparison with prior studies and Official B v1 as a complementary homology-aware evaluation.

These observations highlight a practical difficulty in constructing leakage-resistant benchmarks for PPI prediction, where homologous relationships and complex-level dependencies are often highly interconnected.

### 3.4. Practical Use Case and Unbound-Structure Evaluation

The unbound-structure evaluation was included to better reflect the intended use case, in which the bound complex structure is not available. In this setting, unbound receptor and ligand structures were used as model inputs, while interface labels were derived from the corresponding bound complexes.

Compared with the bound-structure Official A setting, performance decreased on unbound inputs. The ProtBERT-only baseline achieved slightly higher AUPRC, MCC, and F1-score than the full model, together with a lower false-positive rate. This result suggests that sequence representations may be less sensitive to bound-to-unbound conformational changes, whereas structure-derived descriptors can be affected by changes in residue exposure and local conformation.

Nevertheless, the full model substantially reduced false-positive predictions compared with the md = 0 ablation model, indicating that physicochemical descriptors still helped constrain overprediction when unbound structures were used as input. Taken together, these results support the practical relevance of MD-Transformer for prioritizing interface-prone residues from unbound structures, while also indicating that future training should explicitly include unbound or predicted structures to improve transfer to practical prediction settings.

### 3.5. Limitations and Future Directions

Despite retained predictive capability across settings, the model has limitations. The current physicochemical feature set is limited; future work could incorporate additional residue-level signals such as charge, evolutionary conservation, and local geometric descriptors to better characterize interfaces. This study was evaluated using layered splits on DB5.5, and further validation on independent datasets is required. Although we added a direct ESM2-t12-only baseline and an aligned PeSTo comparison, benchmarking against recent methods remains challenging because current protein-interface predictors differ in task formulation, input requirements, training data, and output granularity. Some methods predict binary PPIs or inter-protein residue-residue contacts, whereas MD-Transformer predicts single-chain residue-level interface probabilities. Therefore, we used ProtBERT-only, ESM2-t12-only, and PeSTo as directly evaluated references under the aligned DB5.5 workflow, while other representative methods were summarized for methodological context. A fully unified benchmark across recent PLM-based and structure-aware predictors remains an important direction for future work. In addition, the external-filtered dataset contains only 10 complexes after homology filtering, and the unbound evaluation remains limited to the DB5.5 Official A test complexes. Larger independent datasets with both bound and unbound structures will be needed to further evaluate practical generalization.

A further practical limitation concerns the interpretation of B-factor values in user-provided structures. Because MD-Transformer uses B-factor as one of the physicochemical descriptors, the meaning of this feature depends on the source of the input structure. In X-ray structures, B-factors primarily describe atomic displacement, whereas in cryo-EM structures they may also contain contributions from conformational heterogeneity, local map quality, and model-fitting uncertainty. In AlphaFold-predicted structures, the B-factor column usually stores pLDDT confidence scores instead of experimental B-factors. Therefore, applying the full multimodal model directly to AlphaFold PDB files without preprocessing may lead to misinterpretation of this feature. For predicted structures, users should either use a PLM-only setting, such as the ProtBERT-only or ESM2-t12-only baseline, or replace/disable the B-factor channel rather than treating pLDDT as an experimental B-factor.

Training-set-based Z-score normalization reduces scale differences among continuous descriptors, but it cannot resolve differences in the semantic meaning of B-factor fields across structure sources. Future work may explore more robust measures of flexibility or uncertainty to improve consistency across datasets, extend the current framework to additional structural features, and evaluate its applicability to related prediction tasks such as binding-site annotation and mutation effect prediction.

## 4. Materials and Methods

### 4.1. Dataset Construction and Preprocessing

#### 4.1.1. Data Source: DB5.5

We used DB5.5 as the primary data source [23]. Parsing the official complex list yielded 257 records, which were reduced to 253 unique complexes after removing duplicates by protein_id. During reconstruction of the bound-state complexes, all samples were processed successfully except 1TMQ, which could not be merged due to a chain ID conflict. This resulted in 252 bound-state complexes for downstream modeling. After splitting at the chain level, the dataset contained 650 protein chains with a total of 133,739 residue instances. The dataset size and positive-class statistics are summarized in Table 5.

#### 4.1.2. Structure Integration and Interface Definition

In DB5.5, the bound-state receptor and ligand structures are provided as separate files. To enable consistent inter-chain contact analysis across all samples, we merged the two parts of each complex into a single PDB file.

Interface residues were defined using a standard distance-based rule. A residue was assigned as an interface residue with a label of 1 when the minimum distance between any heavy atom in that residue and any heavy atom in the partner chain was smaller than 6 Å. Residues not satisfying this criterion were assigned as non-interface residues with a label of 0 [30]. This contact-based definition is widely used and facilitates comparison with existing structure-based benchmarks.

#### 4.1.3. Feature Extraction and Normalization

We extracted three structure-related physicochemical descriptors for each residue and aligned them with residue-level sequence embeddings. B-factor values were obtained directly from the temperature factor field in the input PDB file and used as a source-dependent structural descriptor related to local flexibility and conformational fluctuation [16,17]. For experimentally determined X-ray structures, B-factors mainly reflect atomic displacement or thermal mobility. For cryo-EM structures, however, B-factor values may also reflect conformational heterogeneity, local map quality, and model-fitting or alignment uncertainty. Therefore, B-factor values should be interpreted according to the source of the input structure rather than as a uniform physical quantity across all structure types.

For AlphaFold-predicted structures, the B-factor column is commonly used to store pLDDT confidence scores rather than experimental B-factors; thus, raw AlphaFold B-factor columns should not be interpreted as experimental mobility values when applying the full MD-Transformer model. *Solvent-accessible surface area* (SASA) was calculated at the residue level using the Shrake–Rupley algorithm to quantify residue exposure [18]. Hydrophobicity values were assigned according to residue type using the Kyte–Doolittle scale to describe hydrophobic and hydrophilic tendencies [19]. To prevent information leakage, all continuous physicochemical features were Z-score normalized using statistics computed from the training set only. The same normalization parameters were applied to the validation and test sets. This procedure avoids introducing test-set distribution information during normalization.

#### 4.1.4. External-Raw and Homology-Filtered External Dataset

To evaluate model behavior on newly released structural entries, we first constructed an external supplementary dataset, termed external-raw, from 17 PDB complexes released after DB5.5. This dataset contained 97 protein chains and 17,457 residues. Because temporal separation does not necessarily ensure sequence-level independence, all external chains were searched against the Official A training chains using MMseqs2. The distribution of best-hit sequence identities is shown in Figure S8, and the complete external-raw entry list and MMseqs2 homology audit are provided in Supplementary Data S1.

To obtain a homology-filtered external set, external-filtered, we removed an entire external complex if any of its chains showed ≥40% sequence identity and ≥80% query coverage to any Official A training chain. This filtering removed 7 of the 17 external-raw complexes and retained 10 complexes comprising 46 chains. The retained external-filtered dataset contained 9000 residues, including 2589 interface residues, corresponding to a positive ratio of 28.7%. The retained and removed external complexes are listed in Supplementary Data S1. The external-filtered dataset was used only for final evaluation and was not involved in model training, checkpoint selection, threshold selection, or recalculation of normalization statistics.

#### 4.1.5. DB5.5 Unbound Test Set

To assess model performance under a more realistic prediction scenario, we constructed an additional unbound test set from the DB5.5 Official A test split. For each test complex, the unbound receptor and ligand structures were used to extract input features, while the interface labels were derived from the corresponding bound complex. Interface residues were defined using the same heavy-atom distance cutoff as in the bound-structure experiments.

Residues between the bound and unbound structures were mapped by global sequence alignment. Only confidently aligned residues with matching amino-acid identities were retained. One chain was excluded because a reliable bound–unbound mapping could not be established. The final unbound test set contained 26 complexes, 64 chains, and 11,712 residues, including 2602 interface residues.

For the unbound evaluation, B-factor, SASA, and hydrophobicity were recalculated from the unbound structures. The model was not retrained or tuned on this test set. The same training-set-only normalization parameters and the fixed validation-selected threshold of 0.56 were used.

### 4.2. Model Architecture

**Figure 5:**
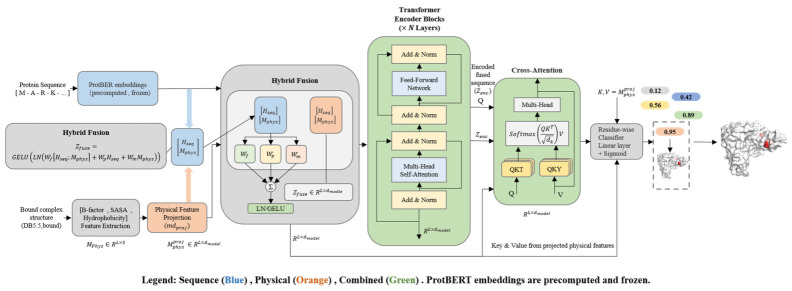
MD-Transformer Framework for Residue-Level PPI Interface Prediction. ProtBERT residue embeddings are precomputed during preprocessing and kept frozen during training. Structure-derived physicochemical descriptors, including B-factor, *solvent-accessible surface area*, and hydrophobicity, were extracted from bound-state complex structures and projected into the hidden feature space. Sequence embeddings and physicochemical features are integrated by the hybrid fusion layer and processed by a multi-layer Transformer encoder to capture contextual dependencies. The encoded representations were then combined with projected physicochemical features through a cross-attention module for cross-modal alignment, and the model produced residue-level interface probability predictions. Red indicates ground-truth interface residues in panel A and higher predicted interface probability in panels B–D, whereas blue indicates non-interface or lower-probability regions.

We propose MD-Transformer, a multimodal model that combines sequence representations with structure-related physicochemical descriptors for residue-level PPI interface prediction. Here, “MD” denotes multi-descriptor, emphasizing joint modeling of multiple residue descriptors. The framework is shown in Figure 5. Residue-level ProtBERT embeddings are precomputed during preprocessing and stored offline; they were kept frozen during training. Physicochemical descriptors were mapped to a shared hidden space through linear projections and are then used for feature fusion and cross-modal interaction.

In the implementation used for all experiments, the ProtBERT residue embedding dimension was 1024, and the physicochemical descriptor dimension was 3, corresponding to B-factor, SASA, and hydrophobicity. The hidden dimension of MD-Transformer was set to 512. The Transformer encoder contained four layers, each with eight attention heads and a feed-forward dimension of 2048. Dropout was set to 0.1. The output layer produced a residue-level sigmoid probability for interface classification.

#### 4.2.1. Multimodal Representations

Given a protein chain of length L, we extracted residue-level contextual embeddings using ProtBERT:

(1) $$H_{seq}\in R^{L\times d_{p}}$$

The physicochemical feature matrix was defined as:

(2) $$M_{phys}\in R^{L\times d_{m}},\;d_{m}=3$$

where the three channels of $M_{phys}$ correspond to B-factor, SASA, and hydrophobicity.

#### 4.2.2. Hybrid Fusion Module

Because sequence embeddings and physicochemical descriptors differ in scale and distribution, we used a hybrid fusion module to align and integrate the two modalities. The fusion layer combines a concatenation-based term with modality-specific projections while preserving information from each modality:

(3) $$Z=GELU\left(LN\left(W_{f}\left[H_{seq};M_{phys}\right]+W_{p}H_{seq}+W_{m}M_{phys}\right)\right)$$

where $\left[H_{seq};M_{phys}\right]$ denotes feature-wise concatenation, $W_{f},\;W_{p}$ and $W_{m}$ are projection matrices for the fused input, sequence features, and physicochemical features, respectively, and *LN* denotes layer normalization.

#### 4.2.3. Encoding and Cross-Modal Interaction

The fused representation (*Z*) was processed by a multi-layer Transformer encoder to model contextual dependencies among residues:

(4) $$X_{enc}=Encoder\left(Z\right)$$

The physicochemical feature matrix was nonlinearly projected to match the model hidden dimension:

(5) $$M_{proj}=\varphi \left(M_{phys}\right)$$

To incorporate structural descriptors after contextual encoding, we applied cross-attention [20,21]. Specifically, $X_{enc}$ serves as the query, while $M_{proj}$ provides the key and value for cross-modal alignment and re-integration:

(6) $$X_{out}=CrossAttn\left(X_{enc},M_{proj},M_{proj}\right)$$

Multi-head attention was used in practice to capture diverse interaction patterns.

#### 4.2.4. Output and Optimization

The output layer converts the enhanced residue representation into an interface probability:

(7) $$P_{i}=\sigma \left(W_{o}h_{i}^{out}+b_{o}\right)$$

Because interface residues are a minority class, we trained the model with weighted binary cross-entropy (weighted BCE):

(8) $$L_{WBCE}=-\frac{1}{N}\sum_{i=1}^{N}\left[\alpha \cdot y_{i}logp_{i}+\left(1-y_{i}\right)log(1-p_{i})\right]$$

where $y_{i}$ is the ground-truth label and α is the positive-class weight computed from the training-set class ratio. The loss was computed only at valid residue positions. Model parameters were optimized with AdamW [31].

### 4.3. Training and Evaluation Settings

#### 4.3.1. Training Strategy

To address class imbalance, we trained the model with weighted BCE and optimized parameters using AdamW. Gradient clipping was applied to improve training stability. Model checkpoints were selected using the validation set: after each epoch, we performed validation inference and chose the checkpoint with the highest validation MCC. This checkpoint was then used for test-set evaluation.

To ensure objective evaluation and prevent information leakage, continuous physicochemical features were Z-score normalized using mean and standard deviation computed on the training set only; the same transformation was applied to the validation and test sets. During inference, a validation-driven threshold selection scheme was used. The optimal decision threshold was selected on the validation set by maximizing the F1-score and subsequently fixed for test-set evaluation. This design keeps threshold selection independent of the test set and avoids inflation from test-time tuning. For external-filtered evaluation, we used the Official A validation-selected threshold of 0.56 and applied the same training-set normalization parameters without external-set-specific tuning.

#### 4.3.2. Evaluation Protocols

Model evaluation was conducted using a layered framework, with the purpose and interpretation of each protocol summarized in Table S10. The two primary DB5.5 protocols were Official A and Official B v1, while CD-HIT/MMseqs2 strict splits, external-filtered evaluation, and unbound-structure evaluation were used as supplementary analyses addressing specific generalization or application scenarios under the Official A protocol, data were split at the protein-complex level, ensuring that chains from the same complex were not assigned to different subsets. This protocol prevents direct complex-level overlap between training, validation, and test sets, but it does not impose an explicit chain-level homology constraint. Official A was therefore used as the main within-benchmark evaluation setting for performance reporting, ablation analysis, structural visualization, and comparison with PeSTo.

To impose stronger homology-related constraints, we further defined the Official B v1 protocol. In addition to complex-level separation, chain-level similarity constraints were introduced using a graph-based clustering strategy based on 3-mer Jaccard similarity. Specifically, each protein sequence was represented by its set of 3-mers, pairwise Jaccard similarity was computed between chains, and chain pairs with Jaccard similarity ≥ 0.30 were connected in the graph. Connected components were then treated as indivisible splitting units to reduce potential leakage caused by homologous chains appearing across different subsets. The 3-mer Jaccard threshold was used as an approximate homology-aware grouping criterion for split construction and was not optimized against model performance. Because this strategy is based on approximate k-mer similarity rather than strict global sequence identity, Official B v1 should be interpreted as a homology-aware split rather than a strict sequence-identity-controlled split.

Supplementary stress-test splits were further constructed using CD-HIT [24] and MMseqs2 [25] at a 40% sequence-similarity threshold. This threshold was used as an interpretable strict homology constraint to examine whether DB5.5 could support sequence-identity-controlled splitting when combined with complex-level separation. As summarized in Table 2 and Table S5, these stricter constraints produced large connected components and substantially altered the intended train/validation/test proportions. Therefore, CD-HIT40 and MMseqs40 were used for dataset connectivity and split-feasibility analysis rather than as the primary model performance protocols.

#### 4.3.3. Baselines and Evaluation Metrics

As an external structure-driven reference, we used PeSTo as a baseline for interface residue prediction [32], with a high-level methodological comparison summarized in Table S9. PeSTo outputs uncalibrated structure-based scores stored in the B-factor field of its output PDB files. To enable residue-level comparison under the Official A protocol, we aligned PeSTo scores to ground-truth labels as follows: within each chain, residues were ordered by their within-chain sequence position and assigned a within-chain index; PeSTo scores were then matched to labels using the complex identifier, chain identifier, and within-chain position as keys. We computed metrics only on residues covered by both methods. This comparison provides an external baseline under the same split, label definition, and evaluation protocol, and does not aim to reproduce PeSTo’s original training setting. Because PeSTo outputs are not probability-calibrated and differ in scale from the probability outputs of our model, only the threshold-independent metrics AUROC and AUPRC were reported for comparison.

Model performance was assessed using AUROC, AUPRC, MCC, precision, recall, and F1. MCC summarizes prediction quality for both classes [33,34], while AUPRC is more informative under class imbalance [35]. We used MCC and AUPRC as the primary metrics. The ProtBERT-only baseline used the same precomputed ProtBERT residue embeddings as the sequence modality of MD-Transformer but excluded all physicochemical descriptors, hybrid fusion, and cross-modal attention modules. It was trained and evaluated using the same data splits, residue-level labels, checkpoint-selection strategy, and validation-selected threshold procedure as the full model.

To provide an additional recent protein language model-based baseline, we trained an ESM2-t12-only residue classifier using residue-level embeddings from facebook/esm2_t12_35M_UR50D. This baseline used the same train/validation/test splits and residue-level labels as MD-Transformer, but excluded all physicochemical descriptors and multimodal fusion modules. The decision threshold was selected on the validation set by maximizing F1-score and then fixed for test evaluation.

#### 4.3.4. Multi-Seed Robustness

To evaluate sensitivity to random initialization and training stochasticity, we trained the full model under Official A and Official B v1 with three different random seeds, while keeping the data splits fixed. We report the mean and standard deviation of test-set metrics across seeds in Table S3. This experiment was used to confirm the robustness of the main conclusions rather than to perform extensive tuning.

#### 4.3.5. Implementation Details

ProtBERT sequence representations were precomputed during preprocessing and kept frozen during training. We used the pretrained checkpoint Rostlab/prot_bert. For each protein chain, we input the amino acid sequence and extracted residue-level hidden states from the last layer as sequence features. Embeddings corresponding to special tokens were excluded, and residue embeddings were aligned one-to-one with interface labels and physicochemical features according to within-chain residue order. Each chain produced a residue embedding matrix $H_{seq}\in R^{L\times 1024}$.

MD-Transformer used a hidden dimension of 512, with 8 attention heads and 4 Transformer encoder layers. The feed-forward dimension was 2048, and GELU was used as the activation function. The output layer produced interface probabilities using a Sigmoid function. Training used AdamW with an initial learning rate of $1\times 10^{-4}$ and weight decay of $1\times 10^{-4}$, together with a ReduceLROnPlateau learning-rate scheduler. To mitigate class imbalance, the positive-class weight in weighted BCE was computed from the training set as $\alpha =\frac{N_{neg}}{N_{pos}}$. Checkpoint selection and threshold determination followed Section 4.3.1.

All models were implemented in PyTorch (2.12.0+cpu). ProtBERT residue embeddings were precomputed using the pretrained checkpoint Rostlab/prot_bert and were kept frozen during model training. The input PLM embedding dimension was 1024 for ProtBERT. Physicochemical descriptors consisted of B-factor, SASA, and hydrophobicity, giving a descriptor dimension of 3. Continuous physicochemical descriptors were normalized using training-set-only Z-score statistics, and the same normalization parameters were applied to validation, test, external-filtered, and unbound evaluations.

The MD-Transformer hidden dimension was set to 512. The Transformer encoder used four layers, eight attention heads, a feed-forward dimension of 2048, and dropout of 0.1. Models were trained with a batch size of 4 for 60 epochs using AdamW with a learning rate of $1\times 10^{-4}$ and weight decay of $1\times 10^{-4}$. A ReduceLROnPlateau scheduler was used with a reduction factor of 0.5 and a patience of 5 epochs. Gradient clipping was applied with a maximum norm of 1.0.

The training objective was weighted binary cross-entropy. The positive-class weight was computed as the ratio of negative to positive residues in the training set. Model checkpoints were selected according to the highest validation MCC. For threshold-dependent reporting, the decision threshold was selected on the validation set by maximizing F1-score and then fixed for test-set evaluation. Unless otherwise stated, experiments used a random seed of 42. Experiments were conducted in PyTorch on a local workstation equipped with an AMD Ryzen 5 5500U CPU and integrated AMD Radeon Graphics. The code does not rely on CUDA-specific operations, and the software environment and package requirements are provided in the GitHub repository.

## 5. Conclusions

We present MD-Transformer, a multimodal framework for residue-level PPI interface prediction that combines ProtBERT sequence representations with structure-related physicochemical descriptors and integrates them through hybrid fusion and cross-modal attention. On DB5.5 under the Official A protocol, the model achieved an AUPRC of 0.564 and MCC of 0.366. Under the more stringent Official B v1 protocol, it achieved an AUPRC of 0.480 and an MCC of 0.242. Ablation experiments and statistical analyses further indicate that physicochemical features contribute to stable performance improvements.

Strict-split analyses further revealed that, on DB5.5, combining strict homology constraints with complex-level constraints can produce large connected components. This connectivity can distort the intended train/validation/test proportions and highlights a practical challenge for strict generalization evaluation. SASA-stratified analysis further suggests that physicochemical descriptors improve specificity mainly by reducing overprediction on broadly exposed residues, although this benefit is accompanied by reduced recall in some exposure environments. Overall, integrating sequence representations with physicochemical descriptors improved interface residue prediction across multiple evaluation settings. Future work will extend the set of structural features, test generalization on independent datasets, and explore applications of this framework to related tasks.

The homology-filtered external evaluation further showed that the full model retained an advantage after removing direct sequence overlap with the training set. The unbound-structure evaluation provided a more realistic prediction scenario and indicated that physicochemical descriptors can reduce false-positive predictions even when features are extracted from unbound conformations, although PLM-only representations may transfer more stably in this setting. Overall, these analyses support the complementarity of contextual sequence representations and residue-level physicochemical descriptors, while also highlighting the need for larger independent and unbound-structure benchmarks.

## Figures and Tables

**Table 1:** Residue-Level Interface Prediction Performance under Official A and Official B v1.

Protocol	Model	AUROC	AUPRC	MCC	Precision	Recall	F1	Threshold(val)
Official A	Full model	0.783	0.564	0.366	0.467	0.596	0.524	0.56
	Ablation (md = 0)	0.692	0.405	0.158	0.251	0.976	0.400	0.56
	PeSTo (realigned)	0.551	0.264	-	-	-	-	-
Official B v1	Full model	0.700	0.480	0.242	0.370	0.719	0.489	0.48
	Ablation (md = 0)	0.580	0.322	0.079	0.275	0.980	0.429	0.48

Threshold-dependent metrics including MCC, Precision, Recall, and F1-score were computed using a decision threshold selected on the validation set by maximizing the F1-score and subsequently fixed for evaluation on the test set. Because PeSTo outputs are not probability-calibrated, only AUROC and AUPRC are reported for PeSTo. Note: “-” indicates that the corresponding threshold-dependent metric was not evaluated under the same validation-selected threshold procedure.

**Table 2:** Connectivity statistics under strict split protocols with different homology constraints.

Split Protocol	Covered Chains	Homology Clusters	Connected Components	Largest Component (Chains)	Target Test (%)	Actual Test (%)	Role
Official B v1	650	-	129	235	10.0	36.2	Main homology-aware split
CD-HIT 40	648	362	108	262	10.0	40.4	Supplementary stress-test
MMseqs2 40	650	377	115	246	10.0	37.8	Supplementary stress-test

The strict-split protocol builds a graph by combining homology clustering constraints with complex-level constraints and uses connected components as indivisible units. Large connected components cause the test-set proportion to deviate from the target. This statistic is independent of feature normalization and reflects structural properties of the dataset under different constraints.

**Table 3:** Three-Model Comparison on the Homology-Filtered External Dataset.

Setting	AUROC	AUPRC	MCC	Precision	Recall	F1	FPR	Pos_Ratio_Pred
Full model	0.711	0.505	0.271	0.423	0.662	0.516	0.365	0.450
Ablation (md = 0)	0.662	0.456	0.147	0.326	0.856	0.472	0.716	0.756
ProtBERT-only baseline	0.663	0.412	0.163	0.466	0.238	0.315	0.110	0.147

Performance on the homology-filtered external dataset. The external-filtered dataset contained 10 complexes, 46 chains, 9000 residues, and 2589 interface residues after MMseqs2-based homology filtering. Threshold-dependent metrics were computed using the fixed threshold of 0.56 selected on the Official A validation set. No retraining, threshold tuning, or recalculation of normalization statistics was performed on the external-filtered dataset.

**Table 4:** Evaluation on the DB5.5 Official A unbound test set.

Setting	AUROC	AUPRC	MCC	Precision	Recall	F1	FPR	Pos_Ratio_Pred
Full model	0.721	0.479	0.273	0.411	0.489	0.447	0.200	0.264
Ablation (md = 0)	0.688	0.395	0.152	0.245	0.975	0.391	0.859	0.885
ProtBERT-only baseline	0.721	0.502	0.318	0.477	0.455	0.465	0.142	0.212

The DB5.5 Official A unbound test set contains 26 complexes, 64 chains, and 11,712 residues, including 2602 interface residues. The positive residue ratio is 22.22%. All models were evaluated without retraining, using the training-set-only normalization parameters and the fixed threshold of 0.56 selected on the Official A validation set. Values are reported to three decimal places.

**Table 5:** Residue-Level Statistics of the DB5.5 Dataset.

Dataset	Number of Bound-State Complexes	Number of Protein Chains	Total Residues	Interface Residues	Positive Ratio
DB5.5	252	650	133,739	30,560	22.85%

Statistics are based on 252 successfully reconstructed bound-state complexes. Samples are defined at the chain level. Interface residues are defined by a minimum inter-chain heavy-atom distance ≤ 6 Å.

## Data Availability

The source code, training and evaluation scripts, external-filtering files, lightweight result tables, and checkpoint-release instructions are available at GitHub: https://github.com/yangjiahui719-afk/MD_Transformer_PPI (accessed on 13 May 2026). Large derived files, including raw PDB structures, PLM embedding caches, and intermediate NPZ files, are not included in the repository because of size and third-party data restrictions. DB5.5 structures are available from the original benchmark source. The trained model checkpoints are provided through the GitHub release associated with the repository.

## Supplementary Materials

The following supporting information can be downloaded at: https://www.mdpi.com/article/10.3390/ijms27135848/s1.
