# Supplementary material for: MD-Transformer: Multimodal Integration of ProtBERT Embeddings and Physicochemical Descriptors for Protein–Protein Interface Residue Prediction
Source: Int J Mol Sci. 2026 Jun 29;27(13):5848. doi: 10.3390/ijms27135848 (PMC13361990; doi:10.3390/ijms27135848)
Supplement: Supplementary file 1 [file ijms-27-05848-s001.zip › Supplementary Materials.pdf]

Supplementary Figures

Figure S1

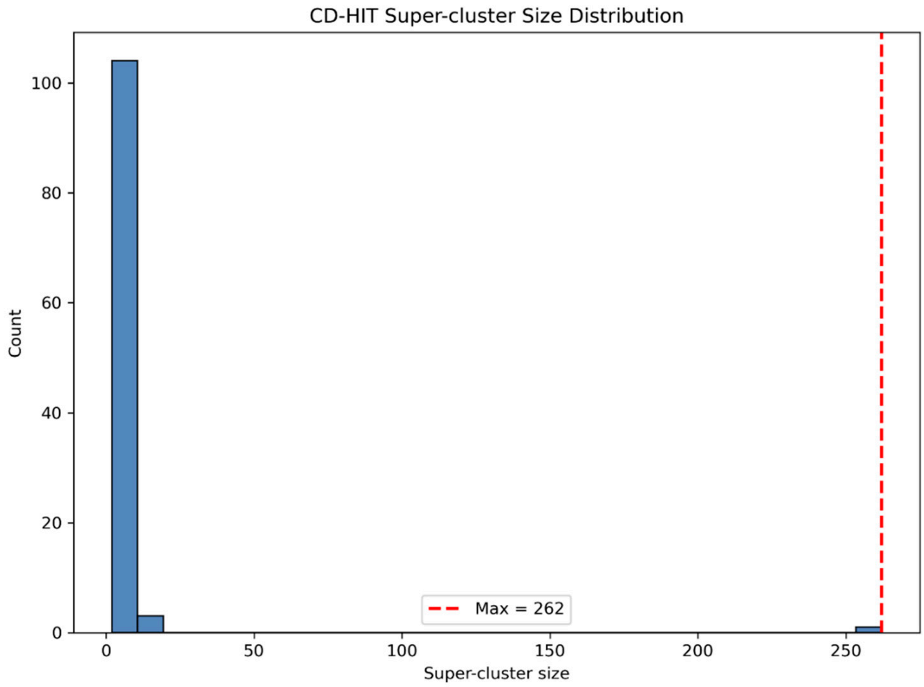

Figure S1. Super-cluster size distribution for the CD-HIT (40%) strict split

Under the combined constraints of CD-HIT clustering at 40% similarity and complex-level non-leakage, chains were treated as graph nodes and edges were added based on homology links; connected components were defined as super-clusters. The resulting histogram shows a long-tailed distribution dominated by a large connected component with a maximum cluster size of 262, leading to substantial deviation of the train, validation, and test proportions from the predefined split targets.

Figure S2

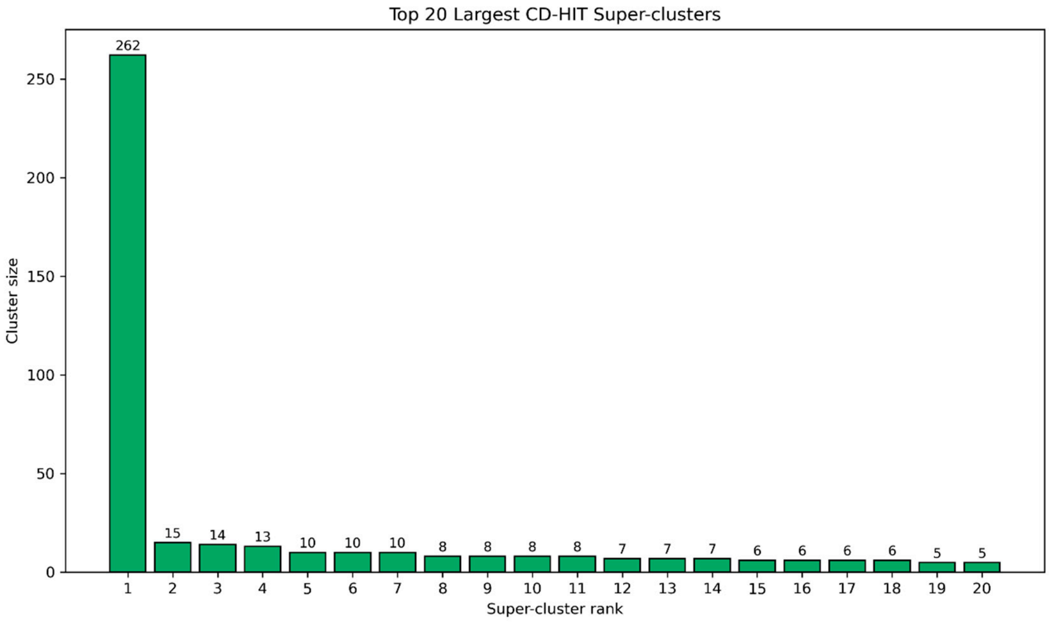

Figure S2. Top 20 largest super-clusters in the CD-HIT (40%) strict split

The bar plot shows the sizes of the 20 largest super-clusters under the CD-HIT strict split. The largest cluster is much larger than the others, indicating that the split-ratio deviation is mainly driven by a small number of giant connected components.

Figure S3

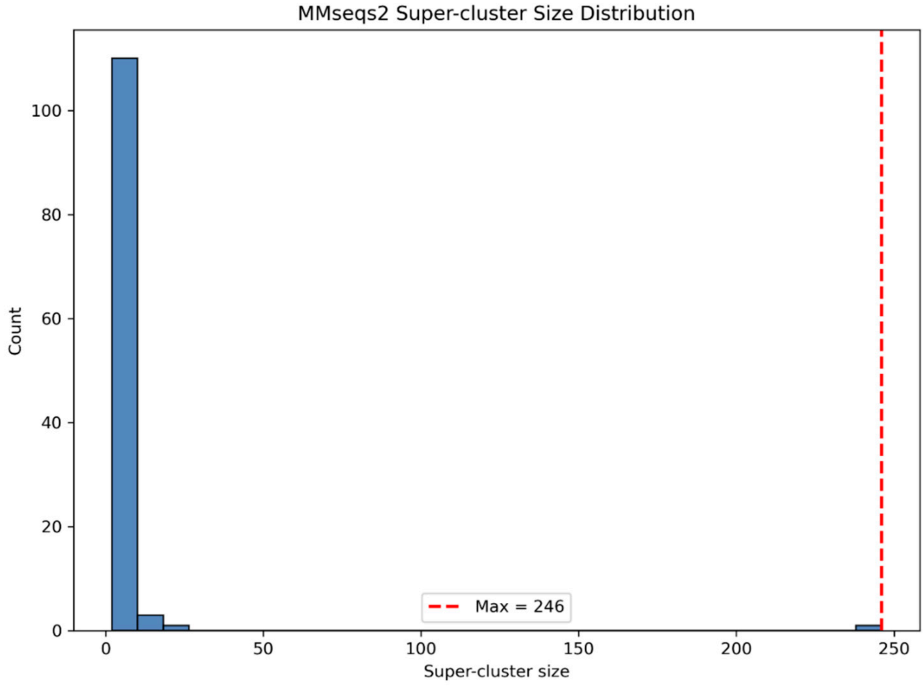

Figure S3. Super-cluster size distribution for the MMseqs2 (40%) strict split

A strict split was constructed using MMseqs2 clustering at 40% sequence similarity together with the complex-level non-leakage constraint. The histogram shows a long-tailed super-cluster size distribution dominated by a large connected component with a maximum cluster size of 246. This pattern is consistent with the distribution observed under the CD-HIT-based strict split.

Figure S4

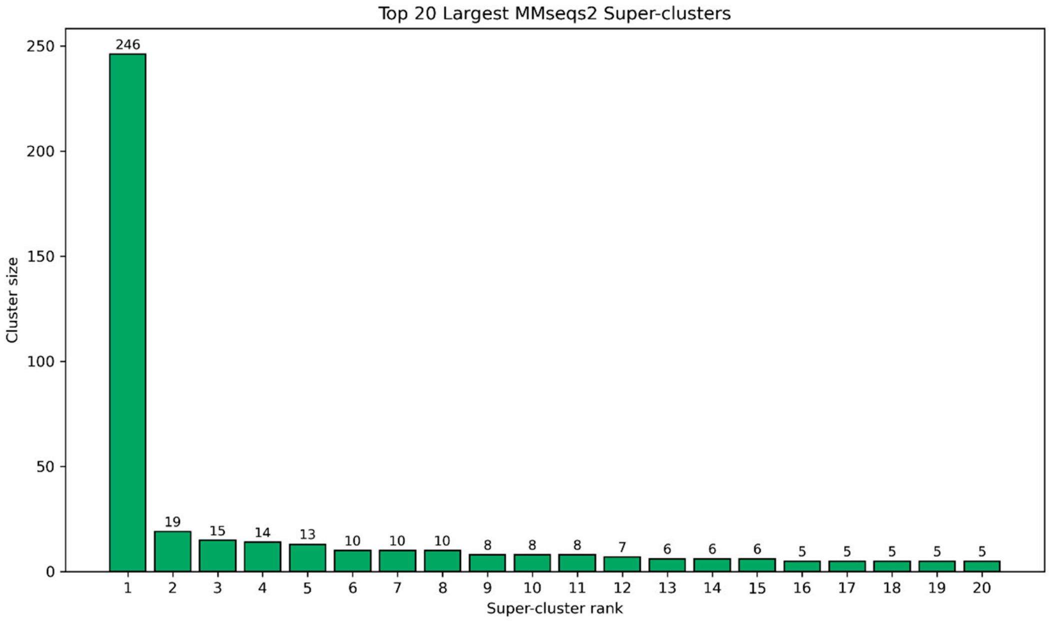

Figure S4. Top 20 largest super-clusters in the MMseqs2 (40%) strict split

The bar plot shows the sizes of the 20 largest super-clusters under the MMseqs2 strict split. The largest cluster greatly exceeds the others, further supporting that DB5.5 readily forms dominant giant connected components when strict homology constraints are combined with complex-level constraints.

Figure S5

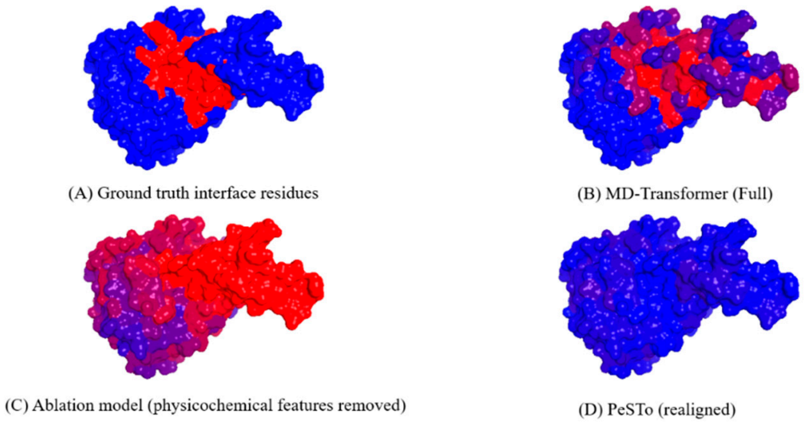

Figure S5. Interface residue prediction visualization for complex 1D6R in the Official A test set

(A) Ground-truth interface residues; (B) MD-Transformer (full); (C) ablation model (physicochemical features set to zero); (D) PeSTo (realigned evaluation). Predicted residue probabilities were written to the B-factor field and visualized in PyMOL.

Figure S6

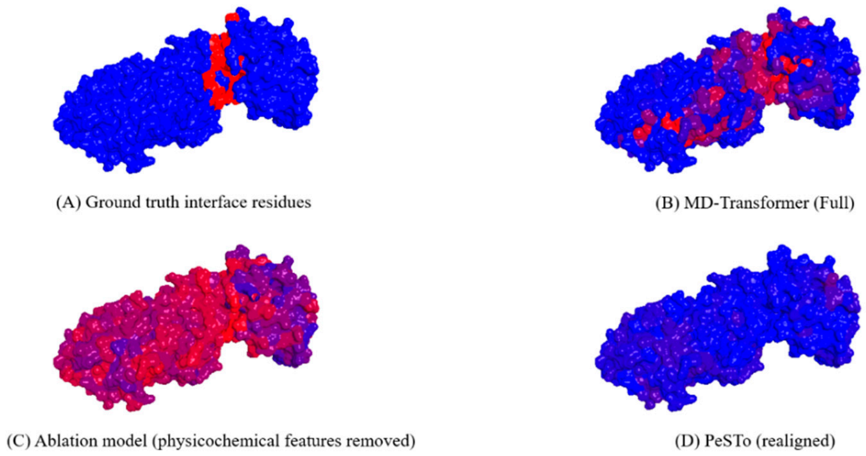

Figure S6. Interface residue prediction visualization for complex 1OPH in the Official A test set

(A) Ground-truth interface residues; (B) MD-Transformer (full); (C) ablation model (physicochemical features set to zero); (D) PeSTo (realigned evaluation). Predicted residue probabilities were written to the B-factor field and visualized in PyMOL.

Figure S7

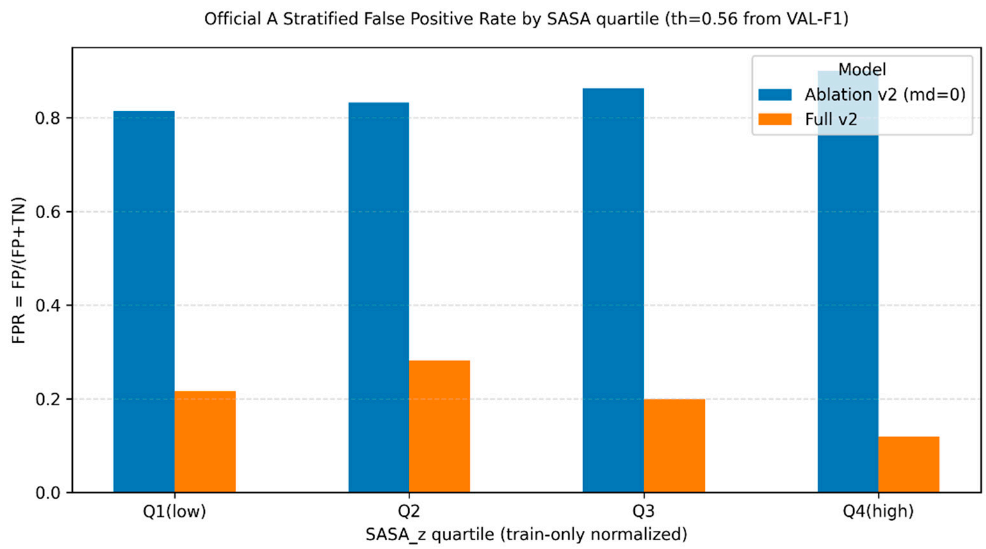

Figure S7. False-positive rate stratified by SASA quartiles under the Official A Setting

SASA values were normalized using Z-score parameters calculated exclusively from the training set. The decision threshold was determined by maximizing the validation F1-score, and the selected threshold of 0.56 was subsequently fixed for test evaluation. The bar plot shows that the ablation model produced a substantially higher false-positive rate among highly exposed residues in the highest SASA quartile than the full model. This result indicates that physicochemical features help suppress false positives on general surface residues.

Figure S8

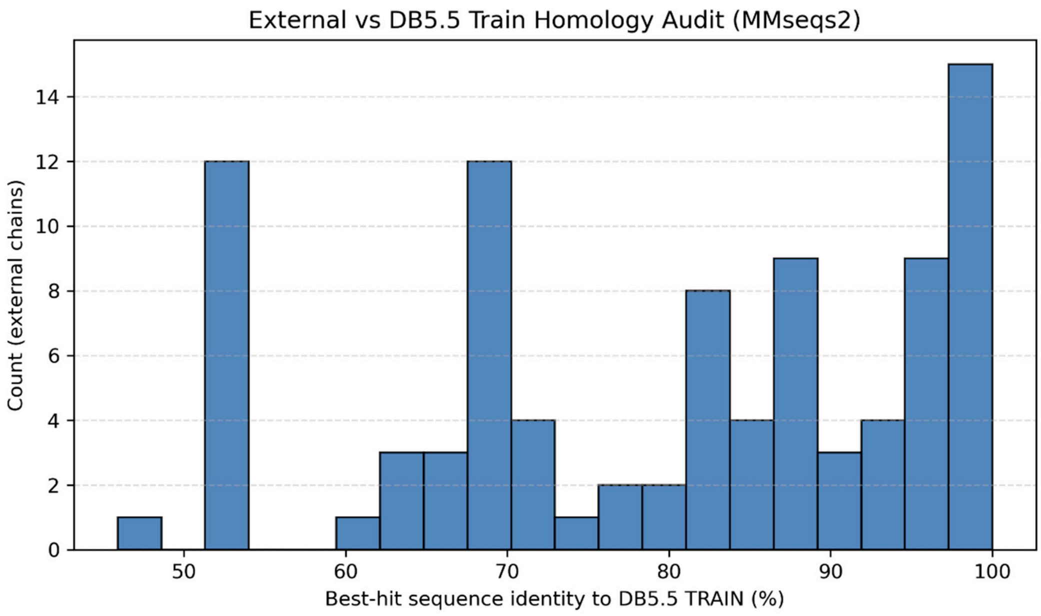

Figure S8. Homology audit of external-raw against the DB5.5 Official A training set

Each chain in external-raw was aligned to the DB5.5 Official A training chains using MMseqs2, and the sequence identity of the best training-set match was recorded. The histogram shows the distribution of best-hit identity and is used to assess homology overlap between external-raw and the training set.

Supplementary Tables

Table S1

Table S1. Paired Wilcoxon signed-rank test of per-complex AUPRC under the Official A setting

Compare	N_proteins	W	p_value	median_diff
Full vs Ablation	26	332	4.57e-06	0.116
Full vs PeSTo	26	348	7.45e-08	0.205

AUPRC was calculated individually for each protein complex. Paired Wilcoxon signed-rank tests were then performed to compare the full model with the ablation model and with PeSTo under the aligned evaluation setting. A one-sided alternative hypothesis was used to test whether the full model achieved superior performance. The table reports the number of complexes, the Wilcoxon test statistic W, the corresponding p-value, and the median difference in AUPRC between the compared methods.

Table S2

Table S2. Sensitivity analysis of interface definition thresholds (6 Å vs. 5 Å) under Official A

r	6Å	6Å	5Å	5Å
Model	Full model	Ablation (md=0)	Full model	Ablation (md=0)
Threshold(val)	0.56	0.56	0.62	0.62
Val_F1(best)	0.527	0.527	0.495	0.495
AUROC	0.783	0.692	0.799	0.691
AUPRC	0.564	0.405	0.546	0.333
MCC	0.366	0.158	0.385	0.190
Precision	0.467	0.251	0.484	0.230
Recall	0.596	0.976	0.529	0.928
F1	0.524	0.400	0.505	0.369
Pos_Ratio	0.227	0.227	0.188	0.188

To evaluate the effect of interface definition, the residue contact distance threshold was changed from 6 Å to 5 Å, and evaluation was repeated under the Official A setting using training-set-only Z-score normalization. Physicochemical features were normalized using train-only Z-score statistics. Threshold-dependent metrics were computed using thresholds selected on the validation set by maximizing the F1-score and then fixed for test evaluation. For consistency, performance metrics for the full and ablation models were calculated on the aligned residue set based on chain position (Pos_In_Chain), with an alignment rate of 1.0. The full model consistently outperforms the ablation model under both definitions, indicating that the main conclusions are robust to the choice of interface threshold.

Table S3

Table S3. Performance stability across multiple random seeds under Official A and Official B v1 settings

Setting	AUROC	AUPRC	MCC	Precision	Recall	F1
Official A	0.7713±0.0140	0.5540±0.0090	0.3573±0.0082	0.4716±0.0208	0.5685±0.0560	0.5138±0.0131
Official B v1	0.6948±0.0048	0.4775±0.0076	0.2379±0.0062	0.3715±0.0085	0.7015±0.0296	0.4855±0.0031

Experiments were conducted using fixed data splits while varying only the random

initialization (n = 3). Physicochemical features were normalized using train-only Z-score statistics. Threshold-dependent metrics were computed using thresholds selected on the validation set by maximizing the F1-score and then fixed for test evaluation. AUROC and AUPRC were calculated directly from prediction probabilities. Results are reported as mean ± standard deviation.

Table S4

Table S4. Alignment coverage of PeSTo predictions under the Official A setting

Protein	full_count	pesto_count	matched_count	match_rate_vs_full	match_rate_vs_pesto
1AVX	395	395	395	1	1
1B6C	433	433	433	1	1
1D6R	281	281	281	1	1
1E4K	604	604	604	1	1
1EER	592	592	592	1	1
1F51	482	482	482	1	1
1FC2	251	251	251	1	1
1JIW	575	575	575	1	1
1K5D	696	696	696	1	1
1OPH	598	598	598	1	1
1SYX	197	197	197	1	1
1XU1	450	450	450	1	1
1Z5Y	254	254	254	1	1
1ZHI	320	320	320	1	1
2AJF	771	771	771	1	1
2NZ8	456	456	456	1	1
2UUY	275	275	275	1	1
2YVJ	508	508	508	1	1
3BX7	293	293	293	1	1
3HI6	606	606	606	1	1
3S9D	307	307	307	1	1
3SZK	404	404	404	1	1
4JCV	993	993	993	1	1
5CBA	300	300	300	1	1
5SV3	292	292	292	1	1
6A0Z	705	705	705	1	1

Residue-level alignment between PeSTo outputs and the test set was performed based on positional indices within each chain (Pos_In_Chain). All 12,038 residues across 26 protein complexes in the Official A test set were successfully matched, yielding a 100% alignment rate. This analysis serves only as an alignment audit and is independent of threshold selection and feature normalization.

Table S5

Table S5. Structural statistics of strict split protocols based on CD-HIT and MMseqs2

Split protocol	CD-HIT (40%)	MMseqs2 (40%)
Covered chains	648	650
Excluded chains	2 (1K74_B,3H11_C)	0

Homology clusters	362	377
Super-clusters	108	115
Max super-cluster	262	246
Top 5 super-cluster sizes	262、15、14、13、10	246、19、15、14、13
Train (%)	39.4	41.5
Val (%)	20.2	20.6
Test (%)	40.4	37.8

The strict split protocol constructs a graph by jointly applying homology clustering constraints and complex-level non-leakage constraints. Connected components, referred to as super-clusters, are treated as indivisible units during dataset partitioning. The table summarizes the number of covered chains, homology clusters, super-clusters, the size of the largest super-cluster, and the resulting train, validation, and test proportions. These statistics are independent of feature normalization and reflect the intrinsic structural characteristics of the dataset under strict partitioning constraints.

Table S6

Table S6. Baseline methods and structural ablation results under the Official A setting

Model	AUROC	AUPRC	MCC	Precision	Recall	F1	Threshold (val)
MD-Transformer (full model)	0.783	0.564	0.366	0.467	0.596	0.524	0.560
MD-Transformer (ablation, md=0)	0.692	0.405	0.158	0.251	0.976	0.400	0.560
Baseline: Simple concat fusion	0.775	0.564	0.353	0.480	0.539	0.508	0.740
Ablation: No Cross-Attention	0.764	0.552	0.356	0.489	0.528	0.508	0.480
Baseline: PhysChem only (3 features)	0.626	0.346	0.161	0.317	0.503	0.389	0.480
ProtBERT-only baseline	0.717	0.503	0.295	0.417	0.547	0.473	0.280

Physicochemical features were normalized using Z-score statistics calculated exclusively from the training set. Threshold-dependent metrics, including MCC, Precision, Recall, and F1-score, were computed using a decision threshold selected on the validation set by maximizing the F1-score through grid search over thresholds ranging from 0.02 to 0.98 with a step size of 0.02. The selected threshold was subsequently fixed for test evaluation. In contrast, AUROC and AUPRC are threshold-independent metrics and were calculated directly from prediction probabilities on the test set. To improve reproducibility, the threshold selection procedure, the optimal validation threshold, and the corresponding validation F1-score are reported. The full model and the physicochemical-feature ablation model were used to evaluate the contribution of multimodal fusion and physicochemical descriptors, respectively.

Table S7

Table S7. Single-feature ablation analysis under Official A with a fixed threshold

Model	Full model	Single-feature ablation: zero SASA	Single-feature ablation: zero B-factor	Single-feature ablation: zero Hydrophobicity
Threshold(val)	0.560	0.560	0.560	0.560
Val_F1(best)	0.527	0.527	0.527	0.527
AUROC	0.783	0.669	0.756	0.764
AUPRC	0.564	0.410	0.542	0.528
MCC	0.366	0.179	0.330	0.345
Precision	0.467	0.288	0.437	0.484
Recall	0.596	0.765	0.582	0.514
F1	0.524	0.418	0.499	0.498
Num_Residues	12038	12038	12038	12038
Delta_AUPRC_vs_Full	0	-0.154	-0.022	-0.036
Delta_MCC_vs_Full	0	-0.188	-0.037	-0.021

Single-feature ablation experiments were performed by individually removing SASA, B-factor, or hydrophobicity while keeping the model parameters and training-set-based Z-score normalization unchanged. Model performance was evaluated on the test set using the fixed threshold of 0.56 selected from the validation set by maximizing the F1-score. Removal of SASA caused the largest performance decrease, with AUPRC decreasing by 0.154 and MCC decreasing by 0.188. This finding indicates that residue exposure information plays an important role in reducing false positives and improving interface localization.

Table S8

Table S8. Stratified false positive analysis based on SASA under the Official A setting

Quartile	Model	N	Pos_Ratio	TN	FP	FN	TP	FPR	Precision	Recall	Threshold (val)	Val_F1 (best)
Q1 (low)	Full	3012	0.246	1781	490	232	509	0.216	0.51	0.687	0.56	0.527
Q1 (low)	Ablatio (md=0)	3012	0.246	420	1851	20	721	0.815	0.28	0.973	0.56	0.527
Q2	Full	3007	0.302	1509	591	295	612	0.281	0.509	0.675	0.56	0.527
Q2	Ablatio (md=0)	3007	0.302	352	1748	26	881	0.832	0.335	0.971	0.56	0.527
Q3	Full	3009	0.206	1915	475	274	345	0.199	0.421	0.557	0.56	0.527
Q3	Ablatio (md=0)	3009	0.206	326	2064	10	609	0.864	0.228	0.984	0.56	0.527
Q4 (high)	Full	3010	0.156	2237	304	303	166	0.12	0.353	0.354	0.56	0.527
Q4 (high)	Ablatio (md=0)	3010	0.156	252	2289	9	460	0.901	0.167	0.981	0.56	0.527

Test residues were stratified into four quartiles according to SASA values normalized using training-set-only Z-score statistics. Test residues were stratified into four quartiles according to

SASA values normalized using training-set statistics, with the fourth quartile representing the highest residue exposure group. Using the fixed decision threshold of 0.56 selected from the validation set, confusion matrix counts together with derived metrics, including false-positive rate, Precision, and Recall, were calculated separately for the full model and the ablation model within each quartile. In the highest SASA quartile, the ablation model produced a false-positive rate of 0.9008, whereas the full model reduced the false-positive rate to 0.1196. These results indicate that physicochemical features, particularly residue exposure information, are important for suppressing false positives among highly exposed surface residues.

Table S9

Table S9. High-level methodological comparison with representative recent interface prediction methods.

Method	Main task	Input	PLM-based	Structure-based	Output	Direct numerical comparison
MD-Transformer	Interface residue prediction	ProtBERT + B-factor /SASA/hydrophobicity	Yes	Yes	Residue-level probability	Yes
ProtBERT-only baseline	Interface residue prediction	ProtBERT embeddings	Yes	No	Residue-level probability	Yes
ESM2-t12-only baseline	Interface residue prediction	ESM2 embeddings	Yes	No	Residue-level probability	Yes
PeSTo	Binding interface prediction	Protein structure	No	Yes	Residue-level score	Yes, aligned
ScanNet	Binding/interface site prediction	Protein structure/geometry	No	Yes	Residue-level score	Not directly rerun
DeepRank-GNN	PPI interface/contact learning	Protein complex graphs	No	Yes	Interface/contact-related score	Not directly rerun

Methodological comparison of the proposed model, PLM-only baselines, and representative recent interface prediction methods. The table summarizes the main task, input modality, output level, and whether each method was directly evaluated under the present DB5.5 residue-level workflow. Numerical comparison was limited to methods whose outputs could be aligned to the same residue-level labels; the remaining methods are included to clarify the benchmarking context.

Table S10

Table S10. Summary of evaluation protocols used in this study

Protocol	Dataset/Input	Splitting or Filtering Strategy	Homology Constraint	Intended Interpretation
Official A	DB5.5 bound structures	Complex-level split; chains from the same complex kept in the same subset	No explicit chain-level homology constraint	Main within-benchmark evaluation, ablation analysis, and PeSTo comparison

Official B v1	DB5.5 bound structures	Complex-level split plus graph-based connected components	Homology-aware grouping using 3-mer Jaccard similarity, k = 3, threshold = 0.30	Reduced-homology generalization test
CD-HIT40/ MMseqs40 stress tests	DB5.5 bound structures	Complex-level constraints combined with sequence-clustering constraints	40% sequence-similarity clustering using CD-HIT or MMseqs2	Dataset connectivity and split-feasibility analysis, not primary performance reporting
External-raw	PDB structures released after DB5.5	Temporal external set	No homology filtering; audited using MMseqs2	Supplementary time-separated evaluation, potentially affected by homology overlap
External-filtered	Homology-filtered subset of external-raw	Remove an entire external complex if any chain has ⩾40% identity and ⩾ 80% query coverage to Official A training chains	MMseqs2-based chain-level filtering	Supplementary external evaluation after reducing direct sequence overlap
DB5.5 unbound test	Unbound structures from Official A test complexes	Unbound receptor/ligand structures used as input; labels retained from corresponding bound complexes	No retraining; same Official A test identities	Practical prediction scenario when bound complex structure is unavailable

summarizes the purpose, splitting or filtering strategy, homology constraint, and intended interpretation of each evaluation protocol used in this study. Official A denotes the primary DB5.5 complex-level split, whereas Official B v1 adds homology-aware grouping based on 3-mer Jaccard similarity. CD-HIT40 and MMseqs40 were used as strict-split stress tests to examine dataset connectivity and split feasibility, rather than as primary performance-reporting protocols. External-filtered and DB5.5 unbound evaluations were used as supplementary tests for homology-filtered external generalization and practical unbound-structure prediction, respectively. No test set was used for training, checkpoint selection, threshold selection, or recalculation of normalization statistics.

Table S11

Table S11. Protein language model baseline comparison under the aligned DB5.5 evaluation workflow

Setting	Model	AUROC	AUPRC	MCC	Precision	Recall	F1
Official A	MD-Transformer (Full)	0.783	0.564	0.366	0.467	0.596	0.524
Official A	ProtBERT-only baseline	0.717	0.503	0.295	0.417	0.547	0.473
Official A	ESM2-t12-only baseline	0.729	0.543	0.299	0.424	0.538	0.474
Official B v1	MD-Transformer (Full)	0.700	0.480	0.242	0.370	0.719	0.489
Official B v1	ESM2-t12-only baseline	0.637	0.408	0.193	0.367	0.581	0.450

compares MD-Transformer with protein language model-only baselines under the same DB5.5 splits, residue-level labels, validation-based threshold selection, and evaluation metrics. The ProtBERT-only baseline used Rostlab/prot_bert embeddings, whereas the ESM2-t12-only baseline

used facebook/esm2_t12_35M_UR50D embeddings. Both PLM baselines excluded physicochemical descriptors and multimodal fusion modules.

Table S12

Table S12. Statistical analysis of predicted interface-residue counts under Official A and Official B v1

Setting	Official A	Official A	Official B v1	Official B v1
Grouping level	Complex (Protein ID)	Chain (Sample_ID)	Complex (Protein ID)	Chain (Sample_ID)
Threshold selected by validation F1	0.56	0.56	0.48	0.48
Number of groups	26	65	77	235
True interface residues	2736	2736	12821	12821
Predicted interface residues	3492	3492	24902	24902
Predicted/true count ratio	1.2763	1.2763	1.9423	1.9423
Total difference, predicted − true	756	756	12081	12081
Mean difference per group	29.0769	11.6308	156.8961	51.4085
Median difference per group	34	8	126	44
Bootstrap 95% CI for mean difference	[7.6154, 48.3471]	[4.9077, 19.0000]	[137.7662, 178.0133]	[45.4595, 57.7959]
Overpredicted groups	23	50	77	223
Underpredicted groups	3	14	0	12
Tied groups	0	1	0	0
One-sided exact sign-test p-value	4.40E-05	3.54E-06	6.62E-24	8.52E-52
One-sided sign-flip permutation p-value	0.00408	0.0004	5.00E-06	5.00E-06

Predicted interface-residue counts were computed using the validation-selected thresholds fixed for test evaluation. Positive differences indicate that the number of predicted interface residues was greater than the number of ground-truth interface residues. Complex-level grouping used protein ID, whereas chain-level grouping used sample ID. The one-sided exact sign test evaluated whether overprediction occurred more frequently than underprediction across groups. The one-sided sign-flip permutation test evaluated whether the group-level predicted-minus-true differences were systematically greater than zero. Bootstrap confidence intervals were computed for the mean difference across groups.
